# Prevalence of obesity and an interrogation of the correlation between anthropometric indices and blood pressures in urban Lagos, Nigeria

**DOI:** 10.1038/s41598-021-83055-w

**Published:** 2021-02-10

**Authors:** Oluseyi Adegoke, Obianuju B. Ozoh, Ifedayo A. Odeniyi, Babawale T. Bello, Ayesha O. Akinkugbe, Oluwadamilola O. Ojo, Osigwe P. Agabi, Njideka U. Okubadejo

**Affiliations:** 1 Department of Medicine, Faculty of Clinical Sciences, College of Medicine, University of Lagos, Idi Araba, Lagos State Nigeria; 2 Department of Medicine, Lagos University Teaching Hospital, Idi Araba, Lagos State Nigeria

**Keywords:** Cardiology, Medical research, Risk factors

## Abstract

Adverse cardiovascular outcomes are linked to higher burden of obesity and hypertension. We conducted a secondary analysis of data for 5135 participants aged ≥ 16 years from our community-based hypertension prevalence study to determine the prevalence of obesity and association between multiple anthropometric indices and blood pressure (BP). The indices were waist circumference (WC), body mass index (BMI), waist-to-height ratio (WHtR), waist-to-hip ratio (WHR), a body shape index(ABSI), abdominal volume index (AVI), body adiposity index (BAI), body roundness index (BRI), visceral adiposity index (VAI) and conicity index (CI). We performed statistical analyses to determine the association, predictive ability, cutoff values and independent determinants of hypertension. Crude prevalence of obesity was 136 per 1000 (95% confidence interval 126–146). BMI had the strongest correlation with systolic and diastolic BP (r_s_ = 0.260 and 0.264, respectively). Indices of central adiposity (AVI, WC, WHtR, BRI) were the strongest predictors of hypertension (≥ 140/90 mmHg), and their cut-off values were generally higher in females than males. WHR, age, BMI and CI were independent determinants of hypertension ≥ 140 mmHg (p < 0.05). We conclude that, based on this novel study, measures of central adiposity are the strongest predictors and independent determinants of hypertension in our population, and cut-off values vary from previously recommended standards.

## Introduction

The Global Burden of Disease (GBD) study provides robust data that cardiovascular diseases (particularly atherosclerotic ischaemic heart disease and stroke) are leading causes of mortality globally, and were responsible for about one-third of all deaths in 2015^1,2^. The inter-regional variation in age-standardized prevalence and death rates reinforces the importance of country-specific data to enhance achievement of the translational outlook of the GBD i.e. drive health policy to mitigate the burden. For instance, age-standardized prevalence rate of cardiovascular diseases for western sub Saharan Africa was the highest (9475 per 100,000) in comparison to the global average of 6403 per 100,000^2^.

Hypertension and obesity are two of the strongest modifiable risk factors for cardiovascular diseases and other non-communicable diseases including type 2 diabetes mellitus. Epidemiological evidence that indicates a persistent linear association of obesity and blood pressures, and the frequent co-existence of obesity and hypertension (particularly in the context of the metabolic syndrome) lends credence to their association^3–5^. In Nigeria, hypertension, overweight or obesity are the major cardiovascular disease risk factors reported, with a high tendency towards co-existence^6^. The mechanistic basis of the obesity-hypertension relationship includes alterations (elevation) of sympathetic nervous system activity, activation of the renin–angiotensin–aldosterone pathway, elaboration of adipokines (e.g. leptin), and hyperinsulinemia^7^.

Population-based interrogation of the relationship between obesity and hypertension is important as such data reinforce existing theories. Additionally, it contributes to understanding the burden and interactions between potentially modifiable primordial factors amenable to public health interventions and can clarify the strength of the associations particularly considering the regional variations and potential genetic, ethnic and geographic contributions.

Several simple and low-cost techniques have been proposed and are variously applied to define adiposity. These include body mass index (BMI), waist circumference (WC) and waist to height ratio (WHtR). In addition, and more recently, anthropometric indices such as body adiposity index (BAI), abdominal volume index (AVI), a body shape index (ABSI), body roundness index (BRI) and conicity index (CI) have been employed as measures of adiposity^8–10^. Not surprisingly, the data addressing their relationship to blood pressure, utility as predictors of other risk factors and cardiovascular outcome is conflicting, and demonstrates outcome specific ethnic and geographic variability^11^. In sub Sahara Africans, variable levels of correlation and remarkable levels of discordance in the relationship between hypertension and some of the anthropometric indices of obesity have been reported^12^.

We conducted a secondary analysis of data from our prospective community-based prevalence of hypertension study carried out in urban Lagos, Nigeria^13^. The aims of this subanalysis were first, to describe the current prevalence of obesity using the body mass index in order to provide a basis for advocating for policies to drive primordial prevention of obesity. Second, to explore the trend of association between various anthropometric indices and blood pressures in our population, and describe the utility of the array of anthropometric measures with respect to predicting hypertension (including determining the measures with greatest magnitude of association). Thirdly, we aimed to derive specific cut-off values for obesity indicators in men and women in the context of our Nigerian population. Ultimately, these data have the potential to direct the application of anthropometric indices in clinical practice and research in our setting.

## Methods

### Study design and participants

We conducted a secondary analysis of data from our prospective community-based prevalence of hypertension study carried out in urban Lagos, Nigeria whose methodology has been previously described^13^. In summary, following ethics approval by the Lagos University Teaching Hospital (LUTH) Health Research Ethics Committee (HREC), we conducted a cross-sectional study utilizing the World Health Organizations STEPwise approach to chronic disease risk factor surveillance (WHO STEPS)^14^. Trained interviewers conducted the door-to-door study of adults aged ≥ 16 years who resided in households within 8 (of 16) randomly selected, densely populated mixed income urban Local Government Areas in Lagos State, Nigeria. The study was conducted between May and December 2017. Written informed consent was obtained from head of household and/or legal guardian and from each individual participant. The study protocol was carried out in accordance with the Declaration of Helsinki.

### Blood pressure and anthropometry

As previously described, blood pressure was measured using a standard protocol—the average of the last two readings out of three measurements taken while seated^14^. The diagnosis of hypertension was based on the JNC 7 criteria^15^. We used the JNC 7 criteria to define hypertension as that was the most widely accepted standard for classification of blood pressures and definition of hypertension at the time of the study. Furthermore, the definition of hypertension as blood pressure of ≥ 140/90 mmHg facilitates comparison of our findings with previous studies. Moreover, the more recent JNC 8 did not address the definitions of hypertension and prehypertension, but rather focused on thresholds for pharmacological treatment^16^. As described in our previous study, persons with previously diagnosed hypertension who were on treatment were regarded as hypertensives even if blood pressure was lower than the cutoff of 140/90 mmHg^13^.

The anthropometric indices were measured with the individuals standing in minimal clothing, with no foot- or head-wears. Weight was measured to the nearest 0.1 kg using a calibrated digital balance while height was measured to the nearest 1 cm on a mobile stadiometer. Waist circumference (WC) was measured to the nearest 0.1 cm at the maximum point of normal expiration mid-way between the lowest rib margin and the iliac crest at the mid-axillary line. Hip circumference (HC) was measured to the nearest 0.1 cm at the level of the maximum extension of the buttocks posteriorly and the pubic symphysis anteriorly^17^. Both WC and HC were measured using a non-stretchable tape placed in horizontal plane. Body mass index (BMI) was calculated as body weight (kg) divided by the square of the height in metre^18^. Weight classification based on BMI (kg/m^2^) was as follows: < 18.5: underweight; 18.5–24.9: normal; 25.0–29.9: overweight; ≥ 30.0: obese {class I: 30.0–34.9; class II: 35.0–39.9; class III: ≥ 40)^19^.

Waist to hip ratio (WHR) was calculated as waist circumference (cm) divided by hip circumference (cm), while waist to height ratio (WHtR) was calculated as waist circumference (cm) divided by height (cm). Other anthropometric indices were derived using the following formulae^20^.

- Body adiposity index (BAI): {hip circumference (cm)/height^1.5^ (m^1.5^)} − 18.
- A body shape index (ABSI): WC (m)/[BMI^2/3^ (kg/m^2^) Height^1/2^ (m)]
- Abdominal volume index (AVI): [2WC^2^ (cm) + 0.7 (WC − HC)^2^ (cm)]/1000
- Body roundness index (BRI): 364.2 − 365.5 [1 − ƛ^−2^ WC^2^ (m) Height^−2^ (m)]^1/2^
- Conicity index (CI): 0.109^−1^ WC (cm) [Weight (kg)/Height (m)]^−1/2^

Normative or cut off values for indicating elevation of each anthropometric index (associated with higher risk of cardiovascular disease) as comparators for data from this study (for male/female where available) are as follows: WC (94 cm/80 cm)^21^; BMI (> 25 kg/m^2^)^19^; WHR (0.90/0.85)^21^; WHtR (0.50)^22^; ABSI (median 0.0744/0.0786)^23^; AVI (14.2 ± 7.1)^24^; BAI (27.1 ± 6.5)^24^; BRI (3.52 ± 1.13/3.86 ± 1.36)^25^*;* CI (1.19 ± 0.17)^24^.

### Data analysis

Data was anonymized and entered in MICROSOFT EXCEL for cleaning before importing into the STATISTICAL PACKAGE FOR SOCIAL SCIENCES (SPSS) version 20.0 for analysis. The data distribution was tested for normality using the Kolmogorov–Smirnov test. Continuous variables are presented as mean ± standard deviation or median (interquartile range) as appropriate. Categorical variables are presented as counts (percentages). Comparisons across gender and between hypertensives and non-hypertensives were performed using student’s t test or Mann–Whitney U test for continuous and chi square for categorical variables. Prevalence of obesity in the study population was derived using the BMI, and expressed as {number in obese category (BMI ≥ 30 kg/m^2^)/total population studied} per 1000 with 95% confidence interval. The age standardized prevalence rate of obesity was calculated based on the WHO World Standard Population 2001^26^.

The relationship between anthropometric indices and blood pressure was first examined using Spearman’s correlation analysis. The receiver operating characteristic (ROC) analysis was used to evaluate the ability of the anthropometric indices to predict hypertension, by calculating the area under curves (AUC) between hypertension and each anthropometric measure. Sensitivity and specificity values for each measure of adiposity were also determined by the receiver operating characteristic (ROC) curves analysis. The optimal cutoff points for WC, WHR, WHtR, BMI, BAI, ABSI, AVI, BRI and CI were established based on the best balance of sensitivity and specificity. Logistic regression analysis was performed to assess for the independent determinants of hypertension. We used the gender specific cut off levels determined by the ROC analysis to categorise all anthropometric measures. The regression model was adjusted for age. Level of significance was set at a two-tailed p value of < 0.05.

### Ethics approval and consent to participate

Ethics approval was obtained from the Lagos University Teaching Hospital (LUTH) Health Research Ethics Committee (HREC). We obtained written informed consent from head of household and/or legal guardian, as well as from each participating individual. The study protocol was carried out in accordance with the Declaration of Helsinki.

## Results

Of the 5578 participants recruited in the study, anthropometric data were available for 5135 (92.1% of study participants) and these were included in the secondary analysis.

### Baseline characteristics and prevalence of obesity

The basic characteristics of the study population stratified by gender are as shown in Table 1. The female to male ratio was 1.08 (2671)–1.0 (2464), with comparable age (37.4 ± 13.3 versus 37.7 ± 12.8; p = 0.36). The combined prevalence of overweight and obesity was 40.1%. Obesity, defined as a BMI ≥ 30 kg/m^2^ was present in 699 (13.6%) of the total population, significantly higher in females (464/2671, 17.4%) compared to males (233/2464, 9.5%) (p < 0.0001). Therefore, crude prevalence rate of obesity in our total population is 136.0 per 1000 (95% confidence interval 126.8–146.0). The total number of our population aged ≥ 18 years was 5095, out of which 695(13.6%) were obese. Thus the crude prevalence rate of obesity in persons aged ≥ 18 years in our urban population was also 136.1 per 1000 (95% confidence interval 126.8–146.0). The distribution of BMI categories in the whole population differed significantly by gender (p < 0.001) and was as follows (male/female respectively): underweight (134 (5.4%)/250 (9.4%)); normal (1447 (58.7%)/1244 (46.6%)); overweight (648(26.3%)/713 (26.7%)); obese (235 (9.5%)/464 (17.4%))—class I (155 (6.3%)/311 (11.6%)), class II (47 (1.9%)/94 (3.5%), class II (33 (1.3%)/59 (2.2%)). The age-standardized prevalence of obesity (per 1000) for persons ≥ 18 years was 87.2 (95% CI 42.8–89.6) overall, 60.9 (26.7–63.5) in males and 111.6 (55.6–115.4) in females.

**Table 1:** Baseline characteristics and anthropometric measurements and indices in the study population.

Characteristics	Total (n = 5135)	Male (n = 2464)	Female (n = 2671)	p values*
Age (years)	37.5 ± 13.1	37.7 ± 12.8	37.4 ± 13.3	0.358
SBP (mmHg)	126.7 ± 18.6	128.9 ± 17.3	124.7 ± 19.6	< 0.001
DBP (mmHg)	80.6 ± 13.2	89.8 ± 13.2	80.4 ± 13.3	0.29
WC (cm)	87.1 ± 13.7	84.4 ± 11.6	89.6 ± 15.0	< 0.001
HC (cm)	98.8 ± 12.9	95.5 ± 10.8	101.7 ± 14.0	< 0.001
BMI (kg/m^2^)	24.6 ± 5.4	24.2 ± 4.9	25.0 ± 5.8	< 0.001
WHR	0.88 ± 0.10	0.89 ± 0.11	0.88 ± 0.10	0.102
WHtR	0.52 ± 0.09	0.49 ± 0.72	0.54 ± 0.09	< 0.001
ABSI (m^7/6^/kg^2/3^)	0.08 ± 0.01	0.08 ± 0.01	0.08 ± 0.01	< 0.001
AVI (cm^2^)	14.95 (6.41)	13.91 (5.27)	15.84 (7.10)	< 0.001
BAI (%)	26.47 (8.49)	24.04 (6.55)	28.71 (8.86)	< 0.001
BRI	3.52 (2.20)	3.06 (1.67)	3.96 (2.35)	< 0.001
CI (m^2/3^/kg^1/2^)	1.25 ± 0.16	1.21 ± 0.14	1.28 ± 0.16	< 0.001

*SBP* systolic blood pressure, *DBP* diastolic blood pressure, *WC* waist circumference, *BMI* body mass index, *WHR* waist-to-hip ratio, *WHtR* waist to height ratio, *ABSI* a body shape index, *AVI* abdominal volume index, *BAI* body adiposity index, *BRI* body roundness index, *CI* conicity index.

*Comparison between gender; data presented as mean ± standard deviation or median (interquartile range).

The anthropometric indices of the participants stratified by gender are shown in Table 1. Females tended to have higher WC, HC, BMI, WHtR, BAI, AVI, BRI and CI (all p < 0.001) but comparable WHR (p = 0.102). Males had significantly higher systolic blood pressure (p < 0.001), but similar diastolic blood pressure (p = 0.29). Compared with those who were non-hypertensive, individuals who were hypertensive had significantly higher anthropometric indices (p < 0.05), (Table 2).

**Table 2:** Comparison of anthropometric indices in hypertensive and non-hypertensive population stratified by gender.

Indices	Hypertensive n = 1402	Non-hypertensive n = 3733	p value
Age (years)	44.6 ± 13.5	34.9 ± 11.9	< 0.001
WC (cm)	92.1 ± 14.4	85.3 ± 12.9	< 0.001
BMI (kg/m^2^)	26.4 ± 5.9	24.0 ± 5.0	< 0.001
WHR	0.90 ± 0.10	0.88 ± 0.10	< 0.001
WHtR	0.55 ± 0.09	0.51 ± 0.08	< 0.001
ABSI (m^7/6^/kg^2/3^)	0.08 ± 0.01	0.08 ± 0.01	0.003
AVI (cm^2^)	16.71 (6.84)	14.18 (5.73)	< 0.001
BAI (%)	28.15 (9.72)	25.81 (8.03)	< 0.001
BRI	4.09 (2.34)	3.30 (1.98)	< 0.001
CI (m^2/3^/kg^1/2^)	1.28 ± 0.16	1.24 ± 0.16	< 0.001

Male (n = 2464)	(n = 704)	(n = 1760)	
Age (years)	43.7 ± 13.2	35.3 ± 11.8	< 0.001
WC (cm)	88.5 ± 12.6	82.8 ± 10.7	< 0.001
BMI (kg/m^2^)	25.6 ± 5.4	23.6 ± 4.6	< 0.001
WHR	0.90 ± 0.11	0.88 ± 0.11	0.006
WHtR	0.52 ± 0.08	0.48 ± 0.07	< 0.001
ABSI (m^7/6^/kg^2/3^)	0.08 ± 0.01	0.08 ± 0.01	0.008
AVI (cm^2^)	15.58 (5.94)	13.40 (4.54)	< 0.001
BAI (%)	25.68 (7.29)	23.47 (6.41)	< 0.001
BRI	3.86 (1.92)	2.87 (1.56)	< 0.001
CI (m^2/3^/kg^1/2^)	1.24 ± 0.15	1.20 ± 0.14	< 0.001

Female (n = 2671)	(n = 699)	(n = 1972)	
Age (years)	45.5 ± 13.7	34.5 ± 11.8	< 0.001
WC (cm)	95.7 ± 15.3	87.5 ± 14.2	< 0.001
BMI (kg/m^2^)	27.2 ± 6.3	24.3 ± 5.4	< 0.001
WHR	0.90 ± 0.09	0.88 ± 0.10	< 0.001
WHtR	0.58 ± 0.09	0.53 ± 0.09	< 0.001
ABSI (m^7/6^/kg^2/3^)	0.08 ± 0.01	0.08 ± 0.01	0.024
AVI (cm^2^)	18.33 (6.95)	15.08(6.74)	< 0.001
BAI (%)	31.22 (9.05)	28.02 (8.16)	< 0.001
BRI	4.80 (2.46)	3.72 (2.17)	< 0.001
CI (m^2/3^/kg^1/2^)	1.32 ± 0.15	1.21 ± 0.16	< 0.001

Data presented as mean ± standard deviation or median (interquartile range). The full data points for ABSI in Hypertensive/Non-hypertensives are, 0.08013 ± 0.0103/0.07915 ± 0.0105, 0.07806 ± 0.01000/0.076919 ± 0.00965 and 0.08221 ± 0.01037/0.08114 ± 0.010847 for total, male and female population respectively.

*SBP* systolic blood pressure, *DBP* diastolic blood pressure, *WC* waist circumference, *BMI* body mass index, *WHR* waist-to-hip ratio, *WHtR* waist to height ratio, *ABSI* a body shape index, *AVI* abdominal volume index, *BAI* body adiposity index, *BRI* body roundness index, *CI* conicity index.

### Association between anthropometric indices and blood pressure

The Spearman’s correlation coefficient for comparison between the various anthropometric indices and blood pressure are shown in Table 3. All the anthropometric indices (except ABSI) had significant strong positive correlation with both systolic and diastolic blood pressures (all p < 0.001). BMI, then AVI and WC had the highest correlation coefficients with both systolic and diastolic blood pressures in females. In males, BMI had the highest correlation coefficient for systolic blood pressure, while AVI followed by WC were the most correlated with diastolic blood pressure.

**Table 3:** Spearman’s correlation of individual anthropometric indices with blood pressure stratified by gender.

Anthropometric indices	Systolic blood pressure		Diastolic blood pressure	
	r_s_	p	r_s_	p
WC (cm)	0.236	< 0.001	0.256	< 0.001
BMI (kg/m^2^)	0.260	< 0.001	0.264	< 0.001
WHR	0.110	< 0.001	0.080	< 0.001
WHtR	0.205	< 0.001	0.230	< 0.001
ABSI (m^7/6^/kg^2/3^)	0.009	0.515	0.031	0.026
AVI (cm^2^)	0.238	< 0.001	0.259	< 0.001
BAI (%)	0.137	< 0.001	0.181	< 0.001
BRI	0.205	< 0.001	0.230	< 0.001
CI (m^2/3^/kg^1/2^)	0.088	< 0.001	0.115	< 0.001
**Male (n = 2464)**				
WC (cm)	0.239	< 0.001	0.236	< 0.001
BMI (kg/m^2^)	0.252	< 0.001	0.218	< 0.001
WHR	0.079	< 0.001	0.084	< 0.001
WHtR	0.235	< 0.001	0.215	< 0.001
ABSI (m^7/6^/kg^2/3^)	0.040	0.047	0.061	0.003
AVI (cm^2^)	0.244	< 0.001	0.238	< 0.001
BAI (%)	0.198	< 0.001	0.159	< 0.001
BRI	0.235	< 0.001	0.215	< 0.001
CI (m^2/3^/kg^1/2^)	0.116	< 0.001	0.129	< 0.001
**Female (n = 2671)**				
WC (cm)	0.291	< 0.001	0.286	< 0.001
BMI (kg/m^2^)	0.293	< 0.001	0.304	< 0.001
WHR	0.131	< 0.001	0.076	< 0.001
WHtR	0.277	< 0.001	0.270	< 0.001
ABSI (m^7/6^/kg^2/3^)	0.042	0.028	0.013	0.498
AVI (cm^2^)	0.292	< 0.001	0.289	< 0.001
BAI (%)	0.225	< 0.001	0.250	< 0.001
BRI	0.277	< 0.001	0.270	< 0.001
CI (m^2/3^/kg^1/2^)	0.114	< 0.001	0.119	< 0.001

*WC* waist circumference, *BMI* body mass index, *WHR* waist-to-hip ratio, *WHtR* waist to height ratio, *ABSI* a body shape index, *AVI* abdominal volume index, *BAI* body adiposity index, *BRI* body roundness index, *CI* conicity index.

### Area under ROC curves (AUC) of the anthropometric indices for predicting hypertension

**Figure 1:**
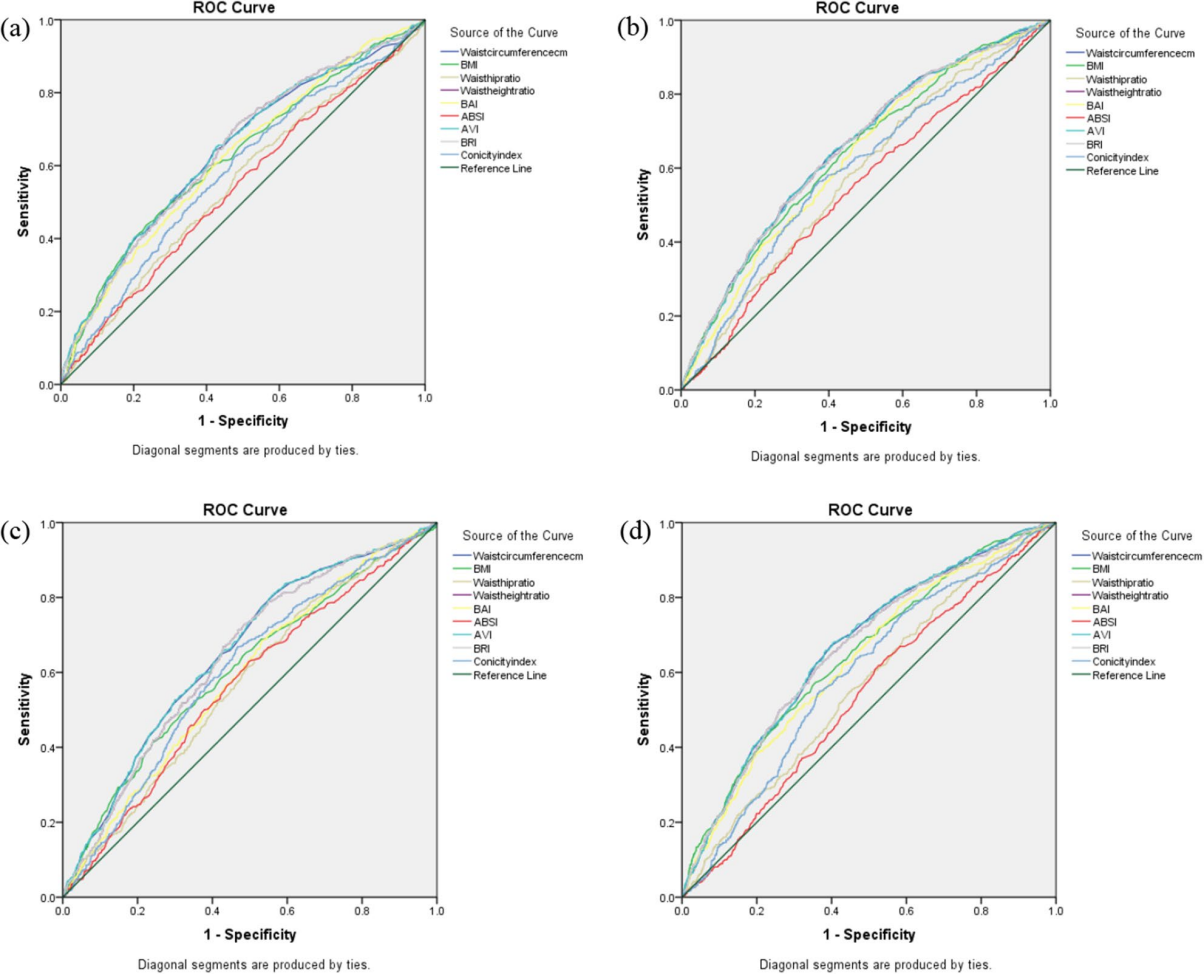
(**a**) ROC for systolic hypertension in males. (**b**) ROC for systolic hypertension in females. (**c**) ROC for diastolic hypertension in males. (**d**) ROC for diastolic hypertension in females.

The area under ROC curve (AUC) for each anthropometric index and hypertension is shown in Table 4. Figure 1 shows the gender-specific ROC curves of the anthropometric indices for predicting hypertension. The AUCs of all the anthropometric indices were larger than 0.5 (p < 0.001) but lower than 0.7 suggesting a moderate predictive significance for hypertension. Overall, AVI and WC showed the largest AUCs of 0.638 and 0.637 for systolic hypertension, with 0.658 and 0.657 for diastolic hypertension, respectively. When stratified by gender, AVI (0.641 (0.614–0.668)) and WC (0.653 (0.626–0.680)) gave the largest AUCs for predicting systolic hypertension in males and females, respectively. AVI and WC had the highest and equal AUCs for diastolic hypertension in males while AVI was the most predictive of diastolic hypertension in females. In both genders and for both systolic and diastolic hypertension, anthropometric indices reflective of central adiposity, (specifically AVI, WC, BRI and WHtR) gave the highest and similar AUCs with overlapping 95% confidence intervals.

**Table 4:** AUCs of anthropometric indices for diagnosing hypertension.

	Systolic hypertension			Diastolic hypertension		
	AUC	95% confidence interval	p value	AUC	95% confidence interval	p value
**Total (N = 5135)**						
WC (cm)	0.637	0.618–0.656	< 0.001	0.657	0.639–0.675	< 0.001
BMI (kg/m^2^)	0.628	0.608–0.647	< 0.001	0.629	0.610–0.648	< 0.001
WHR	0.563	0.544–0.583	< 0.001	0.566	0.547–0.585	< 0.001
WHtR	0.630	0.611–0.659	< 0.001	0.645	0.627–0.663	< 0.001
ABSI (m^7/6^/kg^2/3^)	0.534	0.514–0.554	< 0.001	0.550	0.531–0.569	< 0.001
AVI (cm^2^)	0.638	0.619–0.657	0.001	0.658	0.640–0.676	< 0.001
BAI (%)	0.603	0.584–0.622	< 0.001	0.603	0.584–0.622	< 0.001
BRI	0.630	0.611–0.659	< 0.001	0.645	0.627–0.663	< 0.001
CI (m^2/3^/kg^1/2^)	0.576	0.556–0.595	< 0.001	0.594	0.576–0.613	< 0.001
**Male (n = 2464)**						
WC (cm)	0.638	0.611–0.665	< 0.001	0.652	0.626–0.678	< 0.001
BMI (kg/m^2^)	0.624	0.597–0.652	< 0.001	0.607	0.579–0.635	< 0.001
WHR	0.549	0.521–0.577	0.001	0.569	0.542–0.597	< 0.001
WHtR	0.640	0.613–0.666	< 0.001	0.641	0.615–0.667	< 0.001
ABSI (m^7/6^/kg^2/3^)	0.540	0.512–0.568	0.005	0.565	0.537–0.592	< 0.001
AVI (cm^2^)	0.641	0.614–0.668	< 0.001	0.652	0.626–0.679	< 0.001
BAI (%)	0.622	0.595–0.649	< 0.001	0.587	0.560–0.614	< 0.001
BRI	0.640	0.613–0.666	< 0.001	0.641	0.615–0.667	< 0.001
CI (m^2/3^/kg^1/2^)	0.582	0.555–0.610	< 0.001	0.601	0.574–0.628	< 0.001
**Female (n = 2671)**						
WC (cm)	0.653	0.626–0.680	< 0.001	0.666	0.641–0.690	< 0.001
BMI (kg/m^2^)	0.637	0.610–0.665	< 0.001	0.647	0.622–0.673	< 0.001
WHR	0.578	0.551–0.605	< 0.001	0.563	0.537–0.589	< 0.001
WHtR	0.651	0.624–0.678	< 0.001	0.660	0.635–0.685	< 0.001
ABSI (m^7/6^/kg^2/3^)	0.544	0.516–0.573	< 0.001	0.536	0.510–0.562	< 0.001
AVI (cm^2^)	0.652	0.626–0.679	< 0.001	0.667	0.642–0.691	< 0.001
BAI (%)	0.625	0.598–0.652	< 0.001	0.632	0.607–0.658	< 0.001
BRI	0.651	0.624–0.678	< 0.001	0.660	0.635–0.685	< 0.001
CI (m^2/3^/kg^1/2^)	0.593	0.564–0.621	< 0.001	0.593	0.567–0.618	< 0.001

*AUC* area under curve, *WC* waist circumference, *BMI* body mass index, *WHR* waist-to-hip ratio, *WHtR* waist to height ratio, *ABSI* a body shape index, *AVI* abdominal volume index, *BAI* body adiposity index, *BRI* body roundness index, *CI* conicity index.

### ROC determined cut-off, sensitivity and specificity of anthropometric indices for predicting hypertension

The derived gender specific optimal cut-off points of each anthropometric index that best balanced sensitivity and specificity for systolic and diastolic hypertension are shown in Table 5. In males, BRI and WHtR had the highest sensitivities of 70.1% each for systolic hypertension while BRI (65.9%) was the most sensitive for diastolic hypertension. In females, BAI and WC were most sensitive for systolic (68.1%) and diastolic (69.9%) hypertension. ABSI was the least sensitive but most specific for both systolic and diastolic hypertension. Compared to males, females had higher cut off values of WC, BMI, WHtR, ABSI, BAI, BRI, AVI and CI for predicting both systolic and diastolic hypertension.

**Table 5:** ROC determined cut-off, sensitivity and specificity of each anthropometric index for predicting hypertension.

	Systolic hypertension			Diastolic hypertension		
	Cut-off	Sensitivity (%)	Specificity (%)	Cut-off	Sensitivity (%)	Specificity (%)
**Males (n = 2464)**						
WC (cm)	84.5	64.1	57.9	84.5	64.9	57.7
BMI (kg/m^2^)	23.75	60.5	57.8	24.85	50.3	66.8
WHR	0.8815	58.3	51.9	0.8814	59.5	52.0
WHtR	0.4796	70.1	52.2	0.4927	64.1	58.2
ABSI (m^7/6^/kg^2/3^)	0.0765	58.3	48.4	0.0768	61.5	51.1
AVI (cm^2^)	14.480	64.1	58.1	14.395	65.1	57.5
BAI (%)	24.314	62.4	55.8	23.615	65.1	48.6
BRI	2.985	70.1	52.1	3.184	65.9	57.3
CI (m^2/3^/kg^1/2^)	1.207	60.0	53.5	1.206	65.1	54.1
**Females (n = 2671)**						
WC (cm)	90.5	62.1	61.0	88.95	69.9	55.7
BMI (kg/m^2^)	24.65	63.1	57.3	25.55	56.4	65.1
WHR	0.8856	58.3	54.7	0.8933	51.3	58.1
WHtR	0.5514	60.0	63.0	0.5514	61.0	64.3
ABSI (m^7/6^/kg^2/3^)	0.0845	40.0	68.7	0.0840	38.0	65.6
AVI (cm^2^)	16.497	62.7	60.4	16.563	64.0	62.2
BAI (%)	28.33	68.1	50.8	29.03	62.4	55.9
BRI	4.372	60.0	63.0	4.37	61.0	64.3
CI (m^2/3^/kg^1/2^)	1.309	57.9	60.3	1.311	64.1	63.8

*ROC* receiver operating characteristic curve, *WC* waist circumference, *BMI* body mass index, *WHR* waist-to-hip ratio, *WHtR* waist to height ratio, *ABSI* a body shape index, *AVI* abdominal volume index, *BAI* body adiposity index, *BRI* body roundness index, *CI* conicity index.

### Logistic regression analysis for independent determinant of hypertension

The logistic regression model included all anthropometric measures categorized by gender specific cut off level for systolic hypertension based on the ROC analysis with an adjustment for age (Table 6). We selected the systolic blood pressure cut off levels due to its association with cardiovascular outcome. WHR, age, BMI and CI were independent determinants of hypertension with OR (95% CI) of 1.242 (1.046–1.475), 1.052 (1.047–1.058), 0.707 (0.95–0.841), 0.704 (0.555–0.894), respectively.

**Table 6:** Logistic regression for independent determinants of hypertension (≥ 140/90 mmHg).

Anthropometric indices	Odds ratio	95% confidence interval	p value
WC (cm)	0.686	0.349–1.348	0.274
BMI (kg/m^2^)	0.707	0.595–0.841	< 0.001
WHR	1.242	1.046–1.475	0.014
WHtR	1.090	0.680–1.747	0.720
ABSI (m^7/6^/kg^2/3^)	1.223	0.982–1.523	0.072
AVI (cm^2^)	1.255	0.642–2.454	0.507
BAI (%)	0.861	0.728–1.019	0.081
BRI	0.792	0.488–1.286	0.346
CI (m^2/3^/kg^1/2^)	0.704	0.555–0.894	0.004
Age (years)	1.052	1.047–1.058	< 0.001

Nagelkerke *r*^2^ = 0.185, p < 0.001 for model fit. Model included all gender specific anthropometric cut off levels as determined in the ROC analysis with adjustment for age.

*WC* waist circumference, *BMI* body mass index, *WHR* waist-to-hip ratio, *WHtR* waist to height ratio, *ABSI* a body shape index, *AVI* abdominal volume index, *BAI* body adiposity index, *BRI* body roundness index, *CI* conicity index.

## Discussion

The crude prevalence of obesity documented in urban dwelling adults in this study (13.6%) is indicative of a rise, and is higher than previous reports from the same geographic location for both urban (2002, 8%) and rural (2010, 2%) areas^27,28^. The proportion of persons categorized as overweight (26.5%) similarly represents an increase, giving a combined prevalence of obesity/overweight of 40.1%, higher than the values reported in most earlier studies from Nigeria and across sub Saharan Africa^6,12,29^. The age standardized prevalence rate of obesity in persons ≥ 18 years in our population (using the WHO World Standard Population 2001) was 111.6 per 1000 (95% CI 55.6–115.4) in females and 60.9 per 1000 (95% CI 26.7–63.5) in males^26^. These rates are higher than the 2016 WHO reported age standardized prevalence rate of 46 per 1000 in males and lower than the 131 per 1000 for females reported for Nigeria^30^. This high prevalence of obesity is in consonance with the predictable nutrition transition that is occurring in most low-middle and low-income countries (including Nigeria). Specifically, countries experiencing increased income and access to a more westernized high calorie diet with correspondingly lower activity levels will expectedly enter into a phase characterized by higher burden of obesity and related diseases. Other mechanisms have been implicated, including the interplay between perinatal effectors of metabolism in the setting of maternal malnutrition (metabolic imprinting) which may drive the association between obesity and lower socioeconomic status (SES) and genetic predisposition to obesity^29,31–33^. Increasing trend in obesity in the lower SES has also been observed in other populations, and may be attributable to improvements in healthy lifestyle in higher SES, uninformed high calorie food choices in persons of lower SES, the effect of malnutrition, or a combination of factors^34^. Cultural influences that glorify obesity as mark of affluence, may also have impacted on the population trends towards obesity and overweight in Nigeria as well as other African countries^35,36^.

The higher prevalence of obesity in females compared to males in our study is similar to reports from other African studies and is consistent with the higher contribution of women to the prevalence of obesity in urban West Africa^6,37–39^. This contribution may likely be reinforced by the cultural associations between obesity and beauty among women^40^.

The increasing prevalence of obesity in Nigeria portends an increased risk of cardiovascular and all-cause morbidity and mortality. For example, this study re-asserts the association of obesity with hypertension^6^. Other studies have documented an increase in the burden of other non-communicable diseases (NCDs) such as diabetes, strokes, cancers and other cardiovascular diseases which are largely driven by the presence of obesity and sedentary lifestyles^41^. There is an urgent need for broad-based multi-pronged interventions that include public health education to promote lifestyle modifications that will change the paradigm.

This is a foremost study from Sub-Saharan Africa to evaluate the trends in the association between multiple measures of adiposity and hypertension. We demonstrated that among the indices of obesity assessed, BMI had the strongest correlation with systolic and diastolic blood pressure and was also an independent determinant of hypertension on regression analysis. However, the indices reflective of central adiposity (AVI, WC, WHtR and BRI) were the strongest predictors of systolic and diastolic blood pressure using the ROC analysis. This is important because our findings suggest that measures of central obesity more precisely predict the presence of hypertension compared to BMI (which is more commonly used in clinical practice and research). Similar findings have been reported for the indices of central adiposity (specifically AVI, WC, BRI and WHtR) in Nigeria, other African countries, Asia, and in European populations^12,20,22,24,42–46^. However on regression analysis, WHR had the highest odds ratio (OR) as an independent determinant of hypertension in our population with superiority over BMI. This was also reported in a Korean population but has not been consistent across other populations^37,43,45^. CI a measure of adiposity that is a composite of both central adiposity (WC) and measures of BMI was also a determinant of hypertension, suggesting that both BMI and measures of central adiposity are important contributors to the development of hypertension. Noteworthy is that simple measures such as WC which is noted to predict hypertension may not be an independent determinant. Composite scores that incorporate the WC particularly the WHR therefore may have a greater potential utility in clinical practice. As has been variously reported ABSI had the poorest association and predictive ability for blood pressure and hypertension^25,27,44,47^.

The association between measures of central adiposity and hypertension is plausible based on the mechanistic activity of visceral fat deposits. Infiltration of visceral adipocytes by macrophages triggers the release of pro-inflammatory cytokines with subsequent systemic inflammation, endothelial dysfunction and longitudinal strain on the blood vessels, promoting a pathogenetic basis for hypertension^48,49^. Central adiposity accounts for up to 75% of the risk for hypertension, and neurohumoral and renin-angiotensin mediated triggers resulting in impaired pressure natriuresis, physical compression of the kidneys by peri-renal fat (impairing renal blood flow and further increasing renin production), and increased sympathetic activity (via the effect of leptin and melanocortin) have been implicated^50^. Central adiposity has also been associated with the occurrence of left ventricular hypertrophy, diastolic dysfunction and systolic dysfunction and the consequential adverse cardiovascular effects^51–53^.

Age as a determinant of hypertension has been well documented, and only the WHR had a higher OR for determining hypertension in this study^6,15^. As a non-modifiable risk factor, it underscores the need to intensify efforts aimed at weight management particularly for central adiposity as one ages.

On the basis of our findings (and supported by other prior studies), we reiterate that although the use of BMI as a surrogate of adiposity has become entrenched in medical practice, this may not be the most pragmatic anthropometric approach for determining cardiovascular morbidity and mortality. Whereas BMI does predict cardiovascular events and correlates with BP, it does not specifically identify the highest risk individuals (and highest risk profile) that may require more intense interventions^54,55^.

Therefore, simple measures of central adiposity such as the WHR and CI (which incorporate measures of BMI and WC) which are also easily measured in the clinical setting may be considered as superior alternative measures of adiposity in clinical practice with the added advantage of being more predictive of not just hypertension but overall cardiovascular risk^56,57^. We identified cut-off levels that may be applied in practice to benchmark normality and assess risk of adverse outcomes. Variability in fat distribution based on genetics could play a role in the predictive ability of different measures of adiposity in different racial groups. Standard cut off levels for measures of BMI, WC, and WHR that are in general use were set by the WHO, expert panels and joint task forces, but have been largely based on or adapted from Caucasian or non-black African populations^21,56,58^. As such, the predictive capabilities for cardiovascular risks in other populations will vary somewhat. The cut off levels we found in this present study for WC and WHR in females (90 cm and 0.88, respectively) and males (84.5 cm and 0.88, respectively) differ from those set by the WHO (WC: 94 cm/80 cm, WHR: 0.90/0.85 for males and females, respectively)^21^. The cut off levels we determined for WHtR and BMI in females (0.55 and approximately 25, respectively) were similar to the cut off levels for normality set by the WHO, but lower than those set for males. This deviation from the WHO recommendation is not unusual as ethnic differences have been reported to impact on the appropriate cut off values of anthropometric indices that predict cardiovascular risks^11,59^. Both lower and higher cut off values than the WHO recommended have been variously reported particularly in studies emanating from Asia and some African countries^11,46,59–61^.

Furthermore, in contrast to the WHO set values, but similar to previous reports from Nigeria and Iran, our females had higher cut off values for WC than males^21,37,61^. These disparities from the WHO normal values underpin the value of locally derived cut off levels, or at the least, an awareness of the need to apply lifestyle guidelines to those within close range of set guidelines. Our findings further buttress the sensitivity of anthropometric cut off values to socio-cultural and economic differences. For example, compared to a previous study in Nigeria we obtained slightly lower cut off values for WC and WHtR in males, and WC in females; but higher values for WHtR and BMI in females^37^. This disparity may be explained by differences in the population selection between the two studies. While our population was selected directly from the community irrespective of religion and socioeconomic status, the previous study population was church based. The possibility of the church based population being guided by socio-cultural practices different from those of the general community may not be unlikely.

The major strengths of this study include the population sampling, robust sample size, and the use of door to door recruitment of participants. In addition, the use of multiple measures of obesity enabled us explore and determine the most predictive measures for hypertension and the potential clinical utility of these measures. With the use of robust statistical methods, we demonstrated the variability in cut off levels for some measures of obesity that are distinct from already set values which underscores the need for further research in our population to validate these findings.

We acknowledge that this study is limited in being reflective of the scenario in urban Lagos, where the influences of modernization may be more profound, thus driving the higher prevalence rates documented. However, the city of Lagos is mixed-income, urban and peri-urban, and the most cosmopolitan in Nigeria with representation of nearly all ethnic groups, and the sample is probably representative of the average urban scenario. The study findings should be interpreted in this context with future comparisons based on the population profile.

## Conclusions

The study demonstrates the current relatively high prevalence of obesity and overweight in urban adults in Nigeria, and provides robust data that can be applied to highlight the imperative need to reinforce and implement preventive strategies to change the paradigm.

Furthermore, we demonstrate that both BMI and the anthropometric indices of central adiposity are important contributors to the development of hypertension. However, the anthropometric indices of central adiposity (WHR, CI, AVI, WC, WHtR) may be more predictive of hypertension, and we propose their routine incorporation into clinical practice instead of the over reliance on BMI alone. Thirdly, our findings highlight the relative impreciseness of cutoffs and caution that population specific ranges or cutoff values may be a more practical guide for guiding or benchmarking implementation of lifestyle modifications in practice. We acknowledge that our findings require further corroboration in the context of our population.

## Data Availability

The datasets used and/or analyzed during the current study are available from the corresponding author on reasonable request.

## Abbreviations

ABSI: A body shape index
AUC: Area under curve
AVI: Abdominal volume index
BAI: Body adiposity index
BMI: Body mass index
BRI: Body roundness index
CI: Conicity index
HC: Hip circumference
HREC: Health Research Ethics Committee
LUTH: Lagos University Teaching Hospital
NCDs: Non communicable diseases
ROC: Receiver operating characteristic curve
SD: Standard deviation
SES: Socio economic status
SPSS: Statistical package for social sciences
WC: Waist circumference
WHO: World health organization
WHR: Waist-to-hip ratio
WHtR: Waist to height ratio
